# Acute Adenoviral Gastroenteritis Complicated by Interstitial Edematous Pancreatitis in an Adult Patient: A Rare Clinical Case With a Multidisciplinary Approach

**DOI:** 10.7759/cureus.87328

**Published:** 2025-07-05

**Authors:** Anastasiia K Shkvarok, Yaroslava V Korost, Denys V Reizin, Yehor S Lisovenko

**Affiliations:** 1 Department of Family Medicine, Bogomolets National Medical University, Kyiv, UKR; 2 Department of Surgery, Kyiv City Clinical Hospital №8, Kyiv, UKR

**Keywords:** acute intestinal infection, adenovirus f, adenovirus infection, diarrhea, gastroenteritis, pancreatitis, vomiting

## Abstract

Adenovirus F (type 40/41) is one of the most significant viral etiological agents of acute gastroenteritis in young children. This is a significant and growing cause of pediatric gastroenteritis worldwide, particularly in children under the age of five. However, in rare cases, it may also lead to clinically significant illness in adults. The clinical case we present describes the course of acute adenoviral gastroenteritis complicated with acute pancreatitis and a pronounced intoxication syndrome in a 24-year-old woman from Ukraine.

## Introduction

Adenoviruses are non-enveloped, double-stranded DNA viruses from the *Adenoviridae *family that can cause a wide spectrum of illnesses, including respiratory tract infections, conjunctivitis, cystitis, and gastroenteritis [1, 2]. Among over 50 identified human adenovirus serotypes, types 40 and 41 (species F) are well-established enteric pathogens and are primarily associated with pediatric viral gastroenteritis, particularly in children under two years of age [1-3].

In adults, adenoviral gastroenteritis is uncommon and typically milder. However, certain cases may exhibit severe or prolonged symptoms, especially in individuals with underlying health conditions or after consumption of potentially contaminated food. Serotypes 40/41 are notable for their gastrointestinal tropism and ability to cause non-bloody, watery diarrhea, often with vomiting and low-grade fever [4, 5]. Certain adenovirus types, particularly those classified within subgroup F, are capable of causing not only gastrointestinal manifestations but also severe extraintestinal complications, especially in immunocompromised individuals. These may include hepatitis, hemorrhagic cystitis, interstitial nephritis, and, in rare cases, pancreatitis, reflecting the capacity for systemic involvement beyond the primary site of infection.

This report describes a rare and clinically significant case of acute adenoviral gastroenteritis in an immunocompetent 24-year-old woman, complicated by interstitial edematous pancreatitis. The case is notable for its prolonged course, recurrent symptoms, diagnostic complexity, and the necessity of multidisciplinary care, highlighting that adenoviral infections in adults may be underrecognized and potentially more severe than expected.

## Case presentation

A 24-year-old previously healthy woman from Kyiv, Ukraine, presented to her family doctor with complaints of nausea, mild fever (up to 37.5°C), and watery diarrhea occurring up to five times daily. Her symptoms developed after consumption of homemade tartare prepared with raw beef and eggs. Initial outpatient management included oral rehydration, diosmectite, and symptomatic treatment with paracetamol, along with dietary modifications. However, adherence was partial, and her condition progressively worsened.

By the fourth day of illness, her diarrhea increased to 12 times per day, with green stool. She experienced repeated vomiting and generalized weakness. The patient was evaluated in the emergency department of a private clinic, where hypotension (blood pressure (BP) 90/70 mmHg), signs of dehydration, and abdominal tenderness were noted. She was admitted to the internal medicine ward with a preliminary diagnosis: A09.9 other and unspecified gastroenteritis and colitis of unspecified origin. The differential diagnosis included acute intestinal infection, salmonellosis, acute gastroenteritis, toxic syndrome, and electrolyte imbalance.

The complete blood count (CBC) on the fifth day of illness revealed a normal white blood cell count (5.9 K/μL) with relative neutrophilia (76.14%) and marked lymphopenia (15.22%), resulting in an elevated neutrophil-to-lymphocyte ratio (NLR) of 5.00, suggestive of systemic inflammation (Table 1). Mild metabolic acidosis was present on arterial blood gas analysis (pH: 7.35, base excess (BE): 4.2 mmol/L) (Table 2). A stool polymerase chain reaction (PCR) test confirmed infection with adenovirus serotype 40/41 (adenovirus F), establishing the viral etiology. Other etiological factors underlying the patient's condition were excluded (Table 3).

**Table 1:** Complete blood count of the patient on the fifth day from symptom onset WBC: white blood cells; RBC: red blood cells; NLR neutrophil to lymphocyte ratio; Neu (%): neutrophils (percentage); Neu (K/µL): neutrophils (absolute count); LY (%): lymphocytes (percentage); LY (K/µL): lymphocytes (absolute count); Mon (%): monocytes (percentage); Mon (K/µL): monocytes (absolute count); Eo (%): eosinophils (percentage); Eo (K/µL): eosinophils (absolute count); Bas (%): basophils (percentage); Bas (K/µL): basophils (absolute count); ESR: erythrocyte sedimentation rate

Diagnostics	Result	Reference range
WBC (K/µL)	5.9	4-11
RBC (T/L)	4.64	3.9-5.2
Hemoglobin (g/L)	141	120-156
Hematocrit (%)	40.2	35.5-45.5
Platelet count (K/µL)	261	166-389
Neutrophil to lymphocyte ratio (NLR)	5.00	1.69-3.35
Neu (%)	76.14	40-70
Neu (K/µL)	4.47	1.7-7.2
LY (%)	15.22	20-44
LY (K/µL)	0.89	1.1-4.5
Mon (%)	7.37	2-9.5
Mon (K/µL)	0.43	0.1-0.9
Eo (%)	1.07	0.5-5.5
Eo (K/µL)	0.06	0.02-0.5
Bas (%)	0.20	0-1.75
Bas (K/µL)	0.01	0-0.2
ESR (mm/hour)	3	<15

**Table 2:** Arterial blood gas analysis on the fifth day from symptom onset pH: potential of hydrogen; pCO_2_: partial pressure of carbon dioxide; pO_2_: partial pressure of oxygen; Na+: sodium; K+: potassium; Ca++: ionized calcium; Glu: glucose; Lac: lactate; Hct: hematocrit; pH (T): temperature-corrected pH; pCO_2_ (T): temperature-corrected pCO_2_; pO_2_ (T): temperature-corrected pO_2_; HCO3: bicarbonate; HCO3std: standard bicarbonate; TCO_2_: total carbon dioxide; BEecf: base excess in extracellular fluid; BE(B): base excess in blood; CO_2_c: calculated carbon dioxide content; THbc: total hemoglobin concentration

Diagnostics	Result
Measured 37*C	
pH	7.35
pCO_2_(mmHg)	38
pO_2_(mmHg)	25
Na^+^(mmol/L)	134
К^+^(mmol/L)	3.5
Са^++^(mmol/L)	1.11
Glu (mmol/L)	4.8
Lac (mmol/L)	1.1
Hct (%)	44
Temp-corrected (36.5*C)	
рН (Т)	7.36
pCO_2_ (T) (mmHg)	37
рО_2_ (Т) (mmHg)	24
Derived parameters	
Са^++^(mmol/L)	1.09
HCO3- (mmol/L)	21.0
HCO3std (mmol/L)	20.1
TCO_2 _(mmol/L)	22.2
BEecf (mmol/L)	-4.6
BE(B) (mmol/L)	-4.2
CO_2_c (%)	41
THbc (g/L)	136

**Table 3:** PCR stool panel for gastrointestinal pathogens DNA *Yersinia enterocolitica*, PCR: *Yersinia enterocolitica* (bacterium, DNA detected by PCR); DNA *Shigella* spp., PCR: *Shigella *species (various *Shigella *spp., bacteria, DNA detected by PCR); DNA enteroinvasive *E. coli*, PCR: enteroinvasive *Escherichia coli* (EIEC) (bacterium mimicking shigellosis, DNA detected by PCR); DNA *E. coli* O157, PCR: *Escherichia coli* O157 (enterohemorrhagic *E. coli* strain, bacterium, DNA detected by PCR); DNA *Salmonella *spp., PCR: *Salmonella* species (various *Salmonella *spp., bacteria, DNA detected by PCR); DNA *Campylobacter *spp., PCR: *Campylobacter *species (bacteria, primarily *Campylobacter jejuni* and *Campylobacter coli*, DNA detected by PCR); *Clostridium difficile* toxins (A/B), PCR: *Clostridium difficile* toxins A and B (toxins produced by *Clostridioides difficile*, detected by PCR); *Shiga* toxins (STX1/StX2), PCR: *Shiga *toxins 1 and 2 (toxins produced by *Shigella *spp. and enterohemorrhagic *E. coli*, detected by PCR); RNA norovirus GI, PCR: norovirus genogroup I (virus, RNA detected by PCR); RNA norovirus GII, PCR: norovirus genogroup II (virus, RNA detected by PCR); RNA rotavirus A, PCR: rotavirus group A (virus, RNA detected by PCR); DNA adenovirus F (serotype 40/41), PCR: adenovirus F, serotypes 40 and 41 (enteric adenovirus types causing gastroenteritis, DNA detected by PCR); RNA astrovirus, PCR: astrovirus (virus, RNA detected by PCR); RNA sapovirus (genogroups I, II, III, IV, V), PCR: sapovirus, genogroups I–V (virus, RNA detected by PCR). PCR: polymerase chain reaction

Diagnostics	Result	Reference range
DNA *Yersinia enterocolitica*, PCR	Not detected	Not detected
DNA *Shigella* spp., PCR	Not detected	Not detected
DNA Enteroinvasive *E. coli*, PCR	Not detected	Not detected
DNA *E. coli* O157, PCR	Not detected	Not detected
DNA *Salmonella *spp., PCR	Not detected	Not detected
DNA *Campylobacter *spp., PCR	Not detected	Not detected
*Clostridium difficile* toxins (A/B), PCR	Not detected	Not detected
*Shiga* toxins (stx1/stx2), PCR	Not detected	Not detected
RNA Norovirus G I, PCR	Not detected	Not detected
RNA Norovirus G II, PCR	Not detected	Not detected
RNA Rotavirus A, PCR	Not detected	Not detected
DNA Adenovirus F (serotype 40/41), PCR	Detected	Not detected
RNA Astrovirus, PCR	Not detected	Not detected
RNA Sapovirus (genogroups I, II, III, IV, V), PCR	Not detected	Not detected

Symptomatic treatment was initiated, including intravenous fluids, antiemetics (ondansetron, metoclopramide), probiotics, and dietary adjustments. Despite transient improvement, the patient experienced a relapse upon reintroducing food. On the 11th day of illness, she was readmitted due to severe recurrent symptoms, namely, diarrhea up to 15 times daily, vomiting, and intermittent fever.

**Figure 1:**
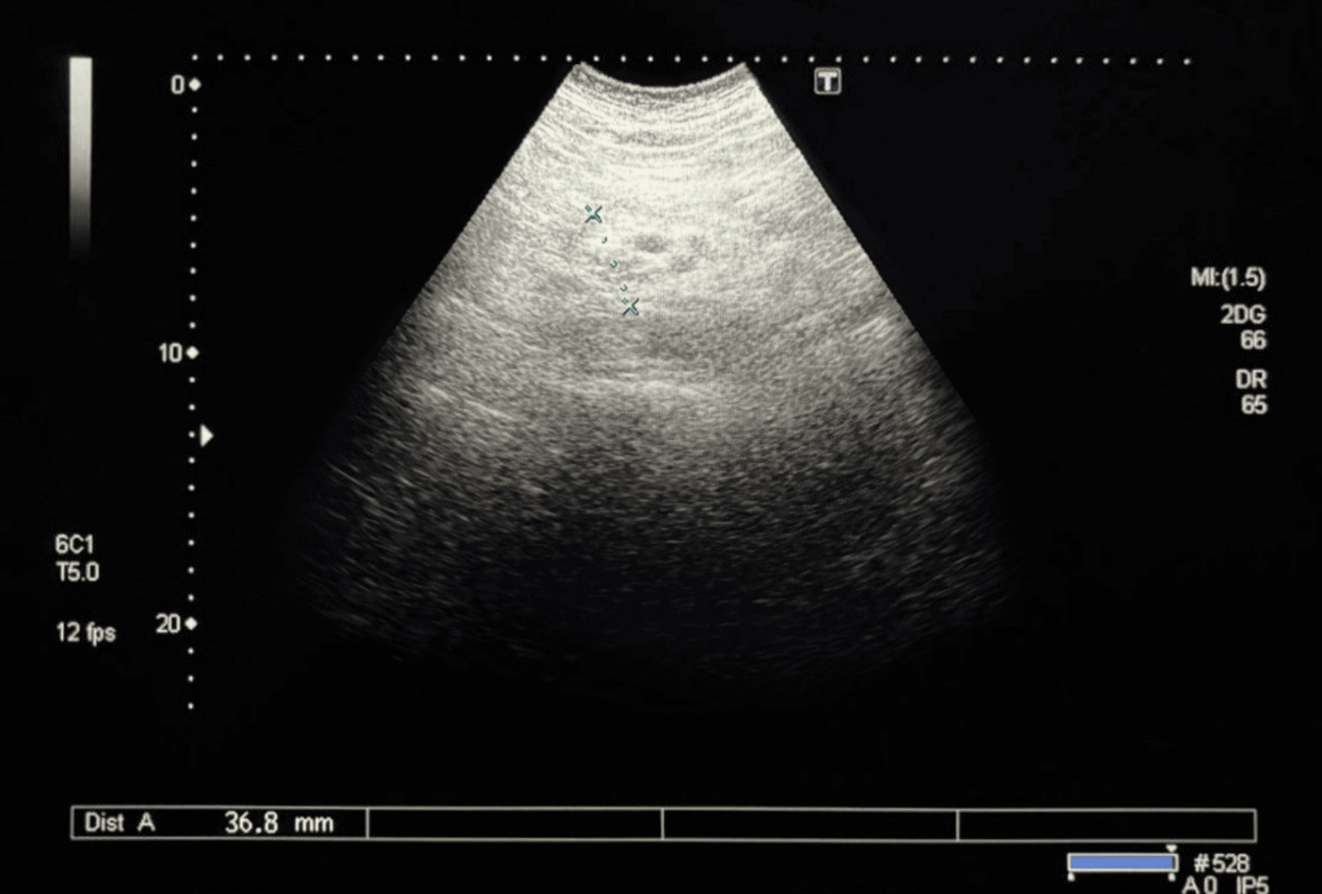
Ultrasound examination of the pancreas (the pancreatic head) The pancreatic head exhibits increased echogenicity with blurred contours, indicative of tissue edema. The structure measures 36.8 mm in transverse section (blue dashed line; Dist A), suggesting volume increase due to the inflammatory process. Surrounding tissues show diffuse decreased echogenicity, typical of edema, with hypoechoic areas that may correspond to inflammatory infiltrates. No distinct signs of necrosis or pseudocysts are present.

**Figure 2:**
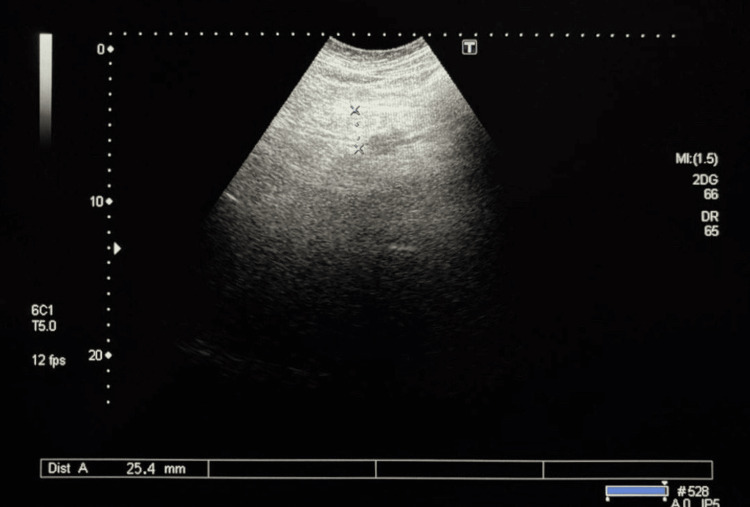
Ultrasound examination of the pancreas (the body of the pancreas) The body of the pancreas is visualized as a homogeneous structure with medium echogenicity and well-defined, smooth contours. The structure measures 25.4 mm in transverse section (blue dashed line; Dist A), which is consistent with the normal range for this anatomical region. The surrounding tissues exhibit normal echogenicity without signs of diffuse changes or focal lesions. No visible pathological inclusions, such as cystic or solid masses, are present.

Further diagnostics revealed no abnormalities in the CBC (Table 4), but elevated serum amylase (96 U/L) was present, raising suspicion of pancreatic involvement (Table 5). Stool antigen testing reconfirmed adenovirus infection, and bacterial stool cultures remained negative. Abdominal ultrasound showed features consistent with interstitial edematous pancreatitis (Figure 1, Figure 2). This confirmed a complication of acute pancreatitis, likely secondary to adenoviral gastroenteritis.

**Table 4:** Complete blood count of the patient from the 11th day of symptom onset WBC: white blood cells; RBC: red blood cells; NLR neutrophil to lymphocyte ratio; Neu (%): neutrophils (percentage); Neu (K/µL): neutrophils (absolute count); LY (%): lymphocytes (percentage); LY (K/µL): lymphocytes (absolute count); Mon (%): monocytes (percentage); Mon (K/µL): monocytes (absolute count); Eo (%): eosinophils (percentage); Eo (K/µL): eosinophils (absolute count); Bas (%): basophils (percentage); Bas (K/µL): basophils (absolute count); ESR: erythrocyte sedimentation rate

Diagnostics	Result	Reference range
WBC (K/µL)	6.25	4-11
RBC (T/L)	4.19	3.9-5.2
Hemoglobin (g/L)	128	120-156
Hematocrit (%)	35.8	35.5-45.5
Platelet count (K/µL)	225	166-389
Neu (%)	63.3	40-70
Neu (K/µL)	3.96	1.7-7.2
LY (%)	25	20-44
LY (K/µL)	1.56	1.1-4.5
Mon (%)	9	2-9.5
Mon (K/µL)	0.56	0.1-0.9
Eo (%)	2.2	0.5-5.5
Eo (K/µL)	0.14	0.02-0.5
Bas (%)	0.5	0-1.75
Bas (K/µL)	0.03	0-0.2
ESR (mm/hour)	6	<15

**Table 5:** Biochemical blood analysis of the patient on the 11th day of illness

Diagnostics	Result	Reference range
Potassium (K⁺) (mmol/L)	3.6	3.5-5.1
Sodium (Na⁺) (mmol/L)	141	135-145
Chloride (Cl⁻) (mmol/L)	105	98-107
Alanine aminotransferase (ALT) (U/L)	22	< 39
Aspartate aminotransferase (AST) (U/L)	18	< 37
Amylase (U/L)	96	13-53
Direct bilirubin (µmol/L)	4	< 5
Indirect bilirubin (µmol/L)	6.6	< 13
Total bilirubin (µmol/L)	10.6	5-21
Creatinine (µmol/L)	82	44-97
Urea (mmol/L)	3.5	2.5-8.3
Blood urea nitrogen (BUN) (mmol/L)	1.64	6.0-20.0
Total protein (g/L)	73	64-83
Albumin (g/L)	46	35-50
C-reactive protein (CRP) (mg/L)	4.5	< 5.0
Blood glucose (mmol/L)	4.3	3.9-5.8

Repeat CBC showed normalization of lymphocyte count (25%) and improved inflammatory markers (C-reactive protein (CRP) 4.5 mg/L). Electrolyte levels were within the reference range. Liver and renal function tests were unremarkable. A comprehensive stool microbiota analysis on day 15 found no pathogenic bacteria or fungi; however, a reduced diversity of commensal flora (*Bifidobacterium *and *Lactobacillus*) was noted (Table 6).

**Table 6:** Results of microbiological stool diagnostics on the 15th day of illness

Diagnostics	Result	Reference range
Bacteriological stool test for dysbiosis	Not detected	0 CFU/mL
Pathogenic Enterobacteriaceae	Not detected	≥ 10⁷ CFU/mL
Bifidobacterium	Not detected	≥ 10⁸ CFU/mL
Lactobacillus	Not detected	10⁶-2×10⁸ CFU/mL
Total *Escherichia coli* (*E. coli*) count	1.28×10⁸ CFU/mL	10⁶-2×10⁸ CFU/mL
*E. coli* with altered enzymatic properties	Not detected	≤ 10⁶ CFU/mL
Lactose-negative *E. coli*	Not detected	≤ 10⁶ CFU/mL
Hemolytic *E. coli*	Not detected	0 CFU/mL
*UPE* (rod- and cocci-shaped forms)	Not detected	≤ 10⁶ CFU/mL
Staphylococcus	Not detected	≤ 10⁴-10⁶ CFU/mL
Hemolytic *Staphylococcus*	Not detected	≤ 10⁴ CFU/mL
*Candida *spp. (yeast fungi)	Not detected	≤ 10⁴ CFU/mL

The patient received multidisciplinary care, including continued intravenous hydration (Ringer’s and 5% glucose), antiemetic and antispasmodic therapy (metoclopramide, drotaverine, papaverine), probiotics (*Saccharomyces boulardii*), pancreatin for enzyme support, and a BRAT diet (short for bananas, rice, applesauce, toast). Clinical improvement began after four days, with decreased diarrhea frequency and resolution of vomiting and fever. She was discharged in stable condition on the 18^th^ day of illness.

## Discussion

This case illustrates a rare but clinically significant course of adenoviral gastroenteritis in an adult patient, complicated by acute interstitial edematous pancreatitis. While the adenoviruses, particularly the serotypes 40 and 41, are primarily associated with enteric infections in children, on occasion, they infect healthy immunocompetent adults with common or severe infections [6].

The virus is most prevalent in African countries, with notable rates also observed in South America and Oceania, indicating a predominance in the Southern Hemisphere [2, 3]. Many factors could have caused the infection, which is not primarily associated with European countries compared to countries in Africa and Oceania. The patient works as a family doctor, and the large number of daily patients and contact with individuals who have recently arrived from abroad increase the risk of infectious exposure. Despite adherence to personal hygiene rules, items such as children's toys for pediatric patients and shared pens used for signing informed consent forms contribute to the spread of infections from patients to the family doctor [7, 8]. Additionally, some declarants arrive from abroad not as tourists but as refugees who have lived in poor hygienic conditions, which also increases the risk of transmission of infectious diseases [9, 10].

Clinical deterioration of the patient despite initial outpatient treatment underscores the potential for rapid progression, especially if oral feeds are reinstated prematurely or hydration is not provided. The diagnosis was established by PCR and antigen detection, which underscores the role of molecular diagnosis in the differentiation of viral from bacterial causes of acute gastroenteritis.

Evidence of pancreatic involvement, confirmed by ultrasound and laboratory findings, suggests that adenoviral infections can elicit gastrointestinal complications, perhaps due to systemic inflammatory reactions or direct viral dissemination [11, 12].

Successful therapy required multidisciplinary coordination, including gastroenterology and infectious disease consultation, and focused on supportive therapy, management of symptoms, and dietary control. The case highlights the importance of early diagnosis, aggressive diagnostic workup, and tailored supportive care even in self-limited viral illness to prevent complications and achieve full recovery.

## Conclusions

Adenoviral gastroenteritis, though more common in pediatric populations, can occur in young adults and may present with atypically severe clinical features, including persistent diarrhea, vomiting, and systemic symptoms. Molecular diagnostic tools (PCR, rapid antigen tests) are crucial for the timely identification of viral pathogens and for avoiding unnecessary antibiotic use in gastrointestinal infections.

Acute interstitial edematous pancreatitis is a rare complication of viral enteric infections, either via systemic inflammatory mechanisms, requiring a high index of suspicion with worsening or recrudescence of gastrointestinal symptoms. Multidisciplinary management and individualized supportive therapy, hydration, antiemetics, diet, and enzyme replacement are paramount in achieving clinical stabilization and recovery. This case illustrates the need for vigilance of clinical dynamics, even in initially mild gastroenteritis cases, to avoid complications and uphold patient safety.
